# *GST* Polymorphisms: Bonassi et al. Respond

**DOI:** 10.1289/ehp.0900838R

**Published:** 2009-09

**Authors:** Stefano Bonassi, Inger-Lise Hansteen, Anna Maria Rossi, Roberto Barale, Lisbeth E. Knudsen, Hannu Norppa

**Affiliations:** Unit of Clinical and Molecular Epidemiology, Istituto di Ricovero e Cura a Carattere, Scientifico San Raffaele Pisana, Roma, Italy, E-mail: stefano.bonassi@sanraffaele.it; Department of Laboratory Medicine, Section of Medical Genetics, Telemark Hospital, Skien, Norway; Department of Biology, Pisa University, Pisa, Italy; Environmental Health, Institute of Public Health, University of Copenhagen, Copenhagen, Denmark; New Technologies and Risks, Work Environment Development, Finnish Institute of Occupational Health, Helsinki, Finland

We thank Vodicka et al. for drawing attention to a critical issue—the role of genetic polymorphisms in the induction of chromosomal damage.

First, we would like to clarify that our study (Rossi et al. 2009) was not designed to evaluate the influence of glutathione *S-*transferase (*GST*) polymorphisms on the frequency of chromosomal aberrations (CAs). Instead, we aimed to identify any possible susceptibility alleles that would increase the strength of the relationship between CAs and risk of cancer in subgroups of genetically variant individuals. The specific purpose of this case–control study explains the relative small size: We planned to obtain data on both CAs and enetic polymorphisms in the same individuals, with all subjects at ages appropriate to allow for an informative follow-up.

The interesting data reported by Vodicka et al. in their letter confirm the extreme heterogeneity of findings when the frequency of chromosome damage is associated with polymorphisms in genes that influence several pathways. In fact, the lack of association in the whole database described by Vodicka et al. contrasts with the significant results reported in exposure-specific subsets, such as the highest frequency of CAs in *GSTT1*-null smokers working in the tire industry (Musak et al. 2008). This interpretation is also supported by the study of Iarmarcovai et al. (2008a), who reviewed 72 population studies reporting a significant effect of *GSTM1*, *EPHX* (microsomal epoxide hydrolase), and *GSTT1* on micronucleus frequency. These authors also reported that the effect observed in specific pathways—such as *ALDH2* in connection to chromosome damage due to alcohol consumption, *BRCA1* and *BRCA2* mutations in breast cancer patients, and *MTHFR* depending on folate levels—was much more pronounced.

A great deal of information about the role of genetic polymorphisms in the induction of chromosomal damage will stem from genome-wide association studies, when enough resources will be available in the field to explore the genetic determinants of chromosome damage using a whole genome scan. Nevertheless, classical association studies are still valid assuming that specific pathways are identified, possibly *a priori*, linking genotoxic exposure to polymorphisms of genes involved in the correspondent pathway

We would also like to address future strategies for exploring the link between the frequency of chromosome damage and the risk of cancer (and other diseases). We fully agree with Vodicka et al.’s recommendation that possible modifying effects of lifestyle, occupational factors, and genetic poly morphisms be taken into account. However, after finding negative results concerning the role of occupational exposure to genotoxic agents and smoking habit (Bonassi et al. 2000), and polymorphisms of metabolic genes (Rossi et al. 2009), we believe that the remaining factor to be investigated is diet. Many studies have reported on the role of various food items and nutrients as modulators of chromosome stability (Fenech 2007); therefore, this parameter now has top priority, although reliable data are difficult to collect retrospectively.

The other recommendation from Vodicka et al. seems less promising, because measuring the frequency of chromosome damage in cancer patients (besides the classical discussion about whether the damage is a cause or a consequence of the disease) is possibly more a marker of progression than a predictive marker (Iarmarcovai et al. 2008b).
